# Day-to-Day Variability of Respiratory Resistance in Asthma and COPD: Influence of Intra-Breath Data Sampling and Observation Period

**DOI:** 10.1109/JTEHM.2026.3653176

**Published:** 2026-01-12

**Authors:** A. Gobbi, C. Gulotta, B. Suki, E. Mellano, M. Vitacca, F. Colombo, V. Brusasco, C. Veneroni, R. Dellacà

**Affiliations:** Restech Srl Milan 20124 Italy; Dipartimento di Area Medica SpecialisticaMalattie Apparato Respiratorio ad indirizzo Fisiopatologico, AOU San Luigi Gonzaga Orbassano (Turin) 10043 Italy; Department of Biomedical EngineeringBoston University 1846 Boston MA 02215 USA; Pneumologia Riabilitativa, Istituti Clinici Scientifici Maugeri IRCCS, Istituto di Lumezzane Brescia 25065 Italy; Pneumologia, Ospedale di Circolo e Fondazione Macchi Varese 21100 Italy; Dipartimento di Medicina SperimentaleUniversità di Genova Genoa 16132 Italy; Dipartimento di ElettronicaInformazione e Bioingegneria, Politecnico di Milano Milan 20133 Italy

**Keywords:** Chronic obstructive pulmonary disease, home monitoring, lung function, oscillometry

## Abstract

Objective: Assessment of lung function variability is recommended for the diagnosis of asthma, but its specificity in separating asthmatic from COPD subjects is low. This study aimed to test the hypothesis that the day-to-day variability of respiratory resistance depends on the respiratory phase considered and observation time. Methods: Respiratory resistance was measured daily by oscillometry at 5 Hz in 47 mild asthmatics, 20 moderate-to-severe COPD, and 35 healthy subjects. The coefficient of variation was calculated over multiple time scales using full breaths, inspiratory phase, or mid-inspiratory phase. Results: The coefficient of variation of mid-inspiratory resistance was significantly higher in asthmatic than healthy and COPD groups at time scales >7 days, but not different between healthy and COPD. The accuracy of the 14-days coefficient of variation of mid-inspiratory resistance in separating asthmatic from the other groups, calculated as the area under the receiver-operating characteristic curve, was 0.86, with 73% sensitivity and 83% specificity at the optimal cutoff of 10%. Moreover, the coefficient of variation was significantly higher in asthma than COPD despite an increased mean resistance in the latter. Conclusion: When expressed as the day-to-day coefficient of variation of mid-inspiratory oscillometric resistance, the variability of lung function does not appear related to the presence or degree of airflow obstruction. Two-week assessment of day-to-day variability of mid-inspiratory resistance provides accurate separation of asthmatic from both healthy and COPD subjects. These findings demonstrate that simple, self-administered daily oscillometry can provide useful clinical information, supporting more accurate asthma diagnosis in real-world settings. Clinical and Translational Impact—The coefficient of variation of mid-inspiratory resistance computed over 14-days separated asthmatic from healthy and COPD subjects with 73% sensitivity and 83% specificity. Daily self-administered oscillometry can support asthma diagnosis.

## I. Introduction

One of the methods to confirm a clinical diagnosis of bronchial asthma is by revealing an excessive day-to-day variability of lung function [1]. A two-week monitoring of peak expiratory flow has been recommended for this purpose, but its sensitivity is moderate and specificity low, particularly in distinguishing between asthma and chronic obstructive pulmonary disease (COPD) [1], [2], [3], [4], [5], [6]. As a consequence, clinicians often face uncertainty when attempting to differentiate asthma from COPD in real-world settings, leading to delays in establishing an accurate diagnosis and initiating appropriate treatment.

In a recent study, we have shown that the variability of respiratory resistance measured by the forced oscillation technique (FOT), also called oscillometry, is more sensitive than peak expiratory flow in separating asthmatics from healthy subjects [6]. However, day-to-day variability of respiratory resistance had also been found elevated in COPD patients [7], thus questioning the assumption that this is an asthma-specific feature rather than simply a corollary to airway narrowing.

One critical issue when assessing the day-to-day variability of respiratory resistance is that neither signal processing nor observation length are standardized. In some studies, resistance was measured over total, i.e., inspiratory and expiratory, breathing cycle [7], [8], [9], [10] in others over the whole- [7] or mid-inspiratory phase [6], [11]. Moreover, the variability of resistance was assessed over minutes [8], [9], [10] or days and defined as standard deviation [7], [8], [9], [10] or coefficient of variation [6], [7], [11]. Whether these technical factors affect the ability of respiratory resistance variability to separate different subjects, e.g., asthmatic from COPD, has not been determined.

In the present study, we hypothesized that the variability of respiratory resistance is critically dependent on the phase and portions of breaths considered, i.e., full breath, full inspiration, or mid inspiration. Therefore, in a first group of subjects, we identified the respiratory phase and observation period that best differentiates asthmatic from both healthy and COPD subjects. We then validated the diagnostic accuracy of day-to-day variability of respiratory resistance and determined its dependence on the degree of airflow obstruction. By identifying clinically optimal measurement conditions and evaluating their diagnostic performance, this study aims to facilitate the translation of oscillometry-derived variability metrics into practical clinical tools capable of supporting differential diagnosis and guiding patient management.

## II. Methods

### A. Subjects and Study Design

The study consisted of two phases and involved 102 adults. The first (development) phase involved 10 well-controlled mild-asthmatic [1], 10 moderate-to-severe COPD [12], and 10 healthy subjects. They self-assessed respiratory input impedance at home by oscillometry for 20 days. The data obtained served to determine the combination of signal processing method and time scale for calculating the variability of resistance that provided the best separation of asthmatics from the other groups.

The second (validation) phase involved 72 additional subjects (37 asthmatics, 10 COPD, and 25 healthy) who took oscillometry measurements at home for 14 consecutive days, which were pooled for analysis with those recorded over the first 2 weeks by the subjects participating in the development phase. These data served to 1) calculate the best cutoff of variability separating the asthmatic from other groups and 2) investigate the relationship between variability and degree of airflow obstruction.

The study was approved by the Ethical Review Boards of each institution where subjects were enrolled, i.e., Ospedale San Luigi (No. 87/2009 and No. 50/2010), Fondazione Maugeri (No. 804 CEC/2012), and Ospedale Luigi Macchi (No. 1058/2012). All subjects gave written informed consent before the start of the study. A subset of oscillometry data used in the second phase was taken from a previously published study [6].

The diagnosis of asthma was based on detailed medical history and confirmed by airway hyperresponsiveness, i.e., a provocative dose of methacholine causing a 20% decrease in FEV ${}_{1}< 1$, $000~\mu $g. [1], [13] Exclusion criteria were smoking history >5 pack-years, obesity (BMI>30 kg/m ${}^{2}$), diabetes, other respiratory diseases, and current regular treatment with inhaled corticosteroids (ICS), or long-acting beta-agonists (LABA), or their combination.

The diagnosis of COPD was based on symptoms, smoking history of >10 pack-years, and confirmed by a post-bronchodilator FEV1/FVC $< 5^{\mathrm {th}}$ percentile of predicted [14]. Exclusion criteria were a post-bronchodilator FEV ${}_{1}> 80$% of predicted, <2 exacerbations in the previous year [15], significant co-morbidities, $\alpha $-1 antitrypsin deficiency, and history of asthma. All COPD patients were under regular treatment with long-acting beta-agonist (LABA), long-acting muscarinic antagonist (LAMA), LABA/LAMA alone or in association with ICS.

Healthy subjects were all never-smokers with a BMI<30 kg/m^2^ and no history of pulmonary disorders.

### B. Measurements

**FIGURE 1.**
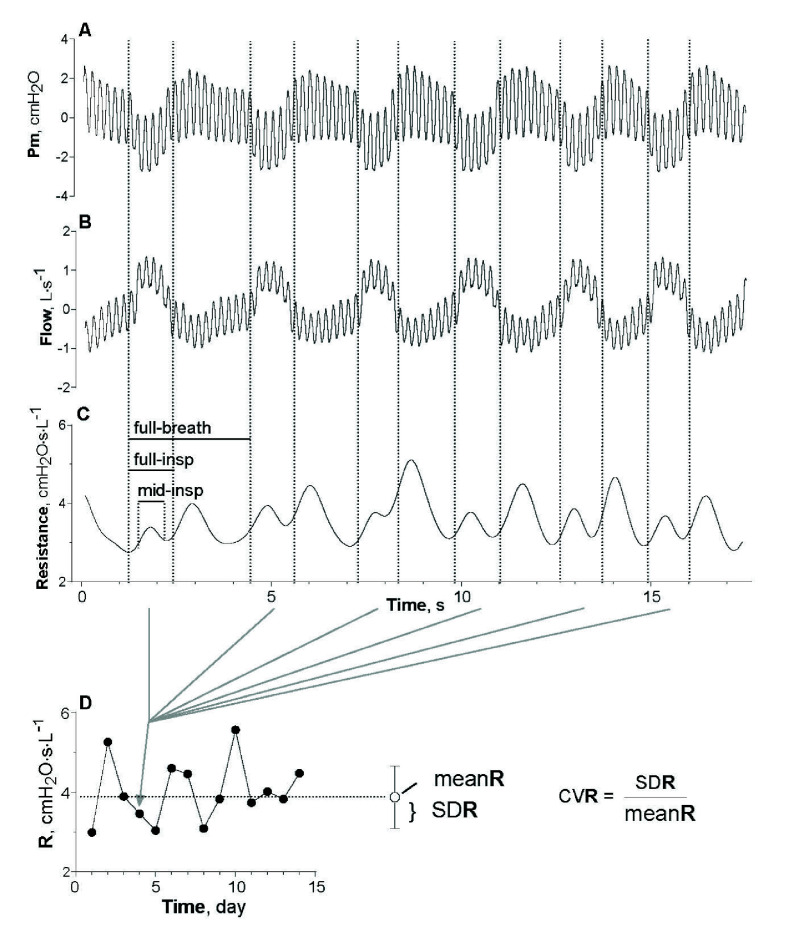
Example of experimental oscillometry tracings and method for calculating the day-to-day variability of respiratory resistance. Mouth pressure (panel A) and airflow (panel B) were recorded for 2 min with a 5-Hz sinusoidal pressure superimposed on tidal breathing. Resistance was calculated using full-breath (R ${}_{\mathrm {full-breath}}$), or full-inspiration (R ${}_{\mathrm {full-insp}}$), or mid-inspiration (R ${}_{\mathrm {mid-insp}}$) of artefact-free tidal breaths (panel C). The resistance data were used for constructing individual daily time-series over the observation period (panel D). The day-to-day variability was defined as the coefficient of variation of resistances (CVR) over a predefined number of days (i.e., a predefined time scale) calculated from standard deviation (SDR) and meanR (panel D).

After enrolment, subjects attended the local pulmonary function laboratory for oscillometry (Resmon PRO DIARY, Restech Srl, Italy), spirometry [16], and lung volumes [17] (Q-Box, Cosmed Srl, Italy) measurements. After appropriate training, each study day in the morning, they collected oscillometry data (Resmon PRO DIARY, Restech Srl, Italy) at home without supervision before taking any medication. Each oscillometry measurement consisted of a 5 Hz sinusoidal pressure forcing applied at the mouth for 2 min, superimposed on spontaneous breathing (Figure 1A-B). A single-frequency waveform was chosen to maximise the signal-to-noise ratio. A frequency of 5 Hz was selected because it is high enough to separate the breathing waveform from the sinusoidal input, yet low enough to retain sensitivity to changes in the lung periphery. Moreover, it ensures comparability with previous studies. The respiratory resistance of each artifact-free breath was computed from flow and pressure signals [18], [19] (Figure 1C) using full breath cycle (R ${}_{\mathrm {full-breath}}$), full inspiration (R ${}_{\mathrm {full-insp}}$), and 20-80% mid-inspiration (R ${}_{\mathrm {mid-insp}}$). After the construction of time-series over the observation period (Figure 1D), the coefficient of variation of different resistance measurements (i.e., CVR ${}_{\mathrm {full-breath}}$, CVR ${}_{\mathrm {full-insp}}$, and CVR ${}_{\mathrm {mid-insp}}$) was calculated for each time scale (i.e., number of consecutive days of data collection), as the ratio between their respective standard deviations (SDR) and mean (meanR).

### C. Statistical Analysis

Between-group differences were tested for significance by ANOVA or Kruskal-Wallis test, as appropriate. Correlation between meanR and the relative SDR and CVR was tested by Pearson correlation. The area under the receiver-operating characteristic curve (AUC) was used to quantify the predictive accuracy of the day-to-day variability of resistance in separating asthmatics from the other groups. An exponential fitting to the AUC values calculated at all time scales was used to determine the time constant ( $\tau $) to reach a plateau (i.e., after $5\cdot \tau $), which was assumed to be the minimum time scale for quantifying variability. The best cutoff was determined in a 5-fold cross-validation procedure on the full pooled dataset. The average of Youden’s index maxima [20] and its performances was reported as sensitivity, specificity, and diagnostic odds ratio [21]. Linear regression data were analysed by a generalized least squares model. To assess the potential impact of age and sex differences among the groups, we performed a Mann–Whitney Rank Sum Test to evaluate differences in parameters between males and females, and a Spearman correlation analysis to examine their associations with age within each group. Prism version 10 (GraphPad Software Inc., CA) was used for all analyses.

## III. Results

### A. Baseline Lung Function

Table 1 summarizes baseline characteristics of the subjects. As per inclusion criteria, FEV_1_ percent predicted and FEV_1_/FVC were markedly lower in COPD than asthmatic (p<0.001 and p<0.05, respectively) and healthy (p<0.001 and p<0.05, respectively) subjects. FEV_1_ percent predicted was also slightly but significantly lower in asthmatic than healthy subjects (p<0.001). Residual volume was significantly higher in COPD than in asthmatic and healthy subjects (p<0.05 for both comparisons), whereas total lung capacity did not differ significantly between groups. R_insp_ in asthmatic subjects was significantly higher than in healthy subjects (p<0.05) but lower than in COPD (p<0.05).

**TABLE 1:** Main Anthropometric and Baseline Lung Function Data

	Development phase			Validation phase		
	Asthma	COPD	Healthy	Asthma	COPD	Healthy
Sex, M/F	7/3	8/2	3/7	26/21	17/3	20/15
Age, yr	35 (15)*	72 (8)*†	47 (11)†	39 (16)*	72 (7)*†	45 (13)†
Weight, kg	71 (10)*	76 (9)†	63 (5)*†	68 (11)	73 (14)	68 (13)
BMI, kg/m^2^	25 (4)	27 (3)	23 (2)	24 (3)	26 (4)	24 (3)
FEV_1_, L	3.00 (0.38)*	0.98 (0.30)*†	3.08 (0.69) †	3.20 (0.93)*	1.05 (0.37)*†	3.58 (0.86)†
FEV_1_, % predicted	87 (11)*‡	37 (11)*†	108 (14)‡†	89 (15)*‡	39 (12)*†	105 (11)‡†
FEV_1_/FVC, % predicted	85 (9)*	40 (11)*†	93 (6)†	92 (10)*	40 (12)*†	100 (13)†
TLC, % predicted	102 (11)	109 (20)	109 (11)	103 (11)	124 (28)	106 (12)
RV, % predicted	109 (34)*	184 (28)*†	103 (20)†	104 (27)*	182 (52)*†	105 (23)†
R_insp_, cmH_2_O⋅ s⋅ L^-1^	3.70 (1.0)*	5.41 (0.78)*†	2.50 (0.40)†	3.55 (0.96)*‡	4.55 (1.06)*†	2.68 (0.53)‡†

Data are mean (SD). BMI: body mass index; FEV_1_: forced expiratory volume in 1 sec; FVC: forced vital capacity; TLC: total lung capacity; RV: residual volume; baseline R_insp_: inspiratory resistance at 5Hz.

^*^ p<0.05 Asthma vs COPD;

^‡^ p<0.05 Asthma vs Healthy;

^†^ p<0.05 COPD vs Healthy. Predicted values for spirometry and lung volumes were from Quanjer et al., 2012 [14] and Quanjer et al., 1993 [28], respectively.

### B. Home Oscillometry Monitoring

#### 1) Quality Control

Adherence to oscillometry measurements was >80% in all groups. At offline quality control, <5% of data were discarded, mostly because of artifacts likely due to forgotten nose-clip or mouthpiece misconnection. No participant dropped out; two asthmatic and two control subjects provided <70% of the expected oscillometry recordings and were not retained for further analyses. None of the asthmatic or COPD subjects experienced exacerbations during the study period.

#### 2) Development Phase

**FIGURE 2.**
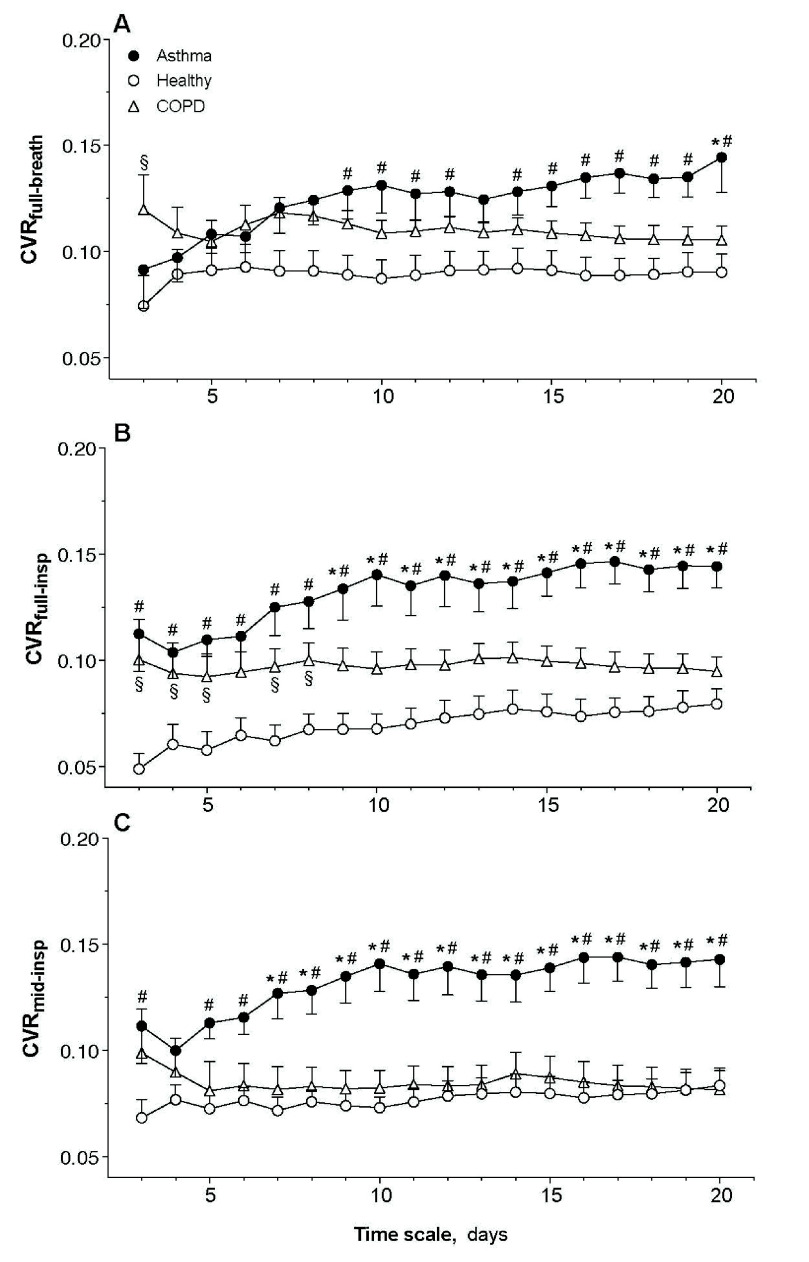
Day-to-day variability of CVR ${}_{\mathrm {full-breath}}$ (panel A), CVR ${}_{\mathrm {full-insp}}$ (panel B), and CVR ${}_{\mathrm {mid-insp}}$ (panel C) as a function of the time scale from asthmatic (full circles), COPD (triangles) and healthy (open circles) subjects recruited for the development phase of the study. Data are reported as mean and standard error. Symbols indicate a statistically significant difference between groups at each time scale with p<0.05. #: Asthma vs. Healthy; $\ast $: Asthma vs. COPD; x: COPD vs. Healthy.

CVR ${}_{\mathrm {full-breath}}$ was significantly higher in asthmatic than healthy subjects with a time scale $\ge 9$ days, but not higher than in COPD (Figure 2A). CVR ${}_{\mathrm {full-breath}}$ in COPD subjects was significantly higher than in healthy subjects only at a time-scale of 3 days. CVR ${}_{\mathrm {full-insp}}$ was significantly higher in asthmatic than healthy subjects at all time-scales and higher than in COPD subjects with time-scale $\ge 9$ days (Figure 2B). In COPD, CVR ${}_{\mathrm {full-insp}}$ was significantly higher than healthy at time scales <9 days, but not thereafter. CVR ${}_{\mathrm {mid-insp}}$ was consistently higher than both healthy and COPD subjects with time-scales $\ge 7$ days (Figure 2C). In COPD, CVR ${}_{\mathrm {mid-insp}}$ was not significantly different from healthy subjects at any time scale.

**FIGURE 3.**
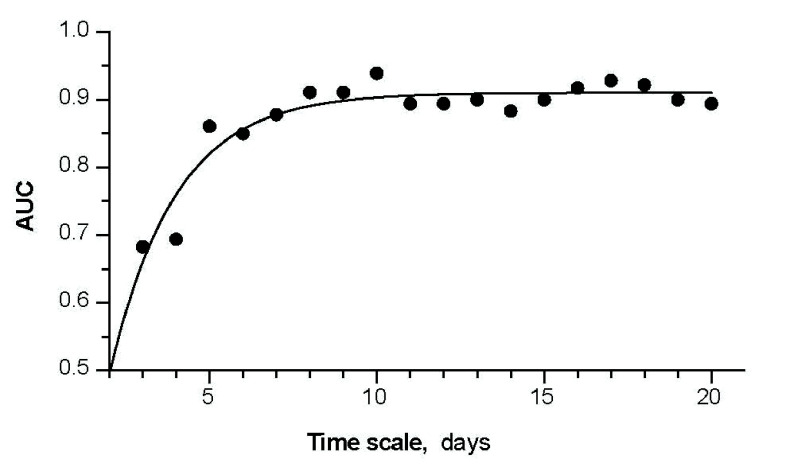
Predictive accuracy (AUC) of CVR ${}_{\mathrm {mid-insp}}$ in identifying asthmatic vs. others as a function of the time scale (full circles). The solid line is the exponential curve fitting the data set. Data is from the development phase of the study.

The accuracy (AUC) of CVR ${}_{\mathrm {mid-insp}}$ for separating asthmatic from both healthy and COPD subjects participating in the development phase increased exponentially (r ${}^{2}=0.88$, $\tau =1.96$) and plateaued at values >0.9 for time scales $\ge 10$ days (Figure 3).

#### 3) Validation of CVR ${}_{Mid-Insp}$ in Identifying Asthmatic Subjects

Based on the results of the development phase, the computation of CVR ${}_{\mathrm {mid-insp}}$ and a two-week observation time were chosen for the second phase of the study. The two-week duration was chosen to allow for up to 4 missing measurements, i.e., approximately 30% missing data per subject, while still providing at least ten data points for CVR ${}_{\mathrm {mid-insp}}$ computation.

**FIGURE 4.**
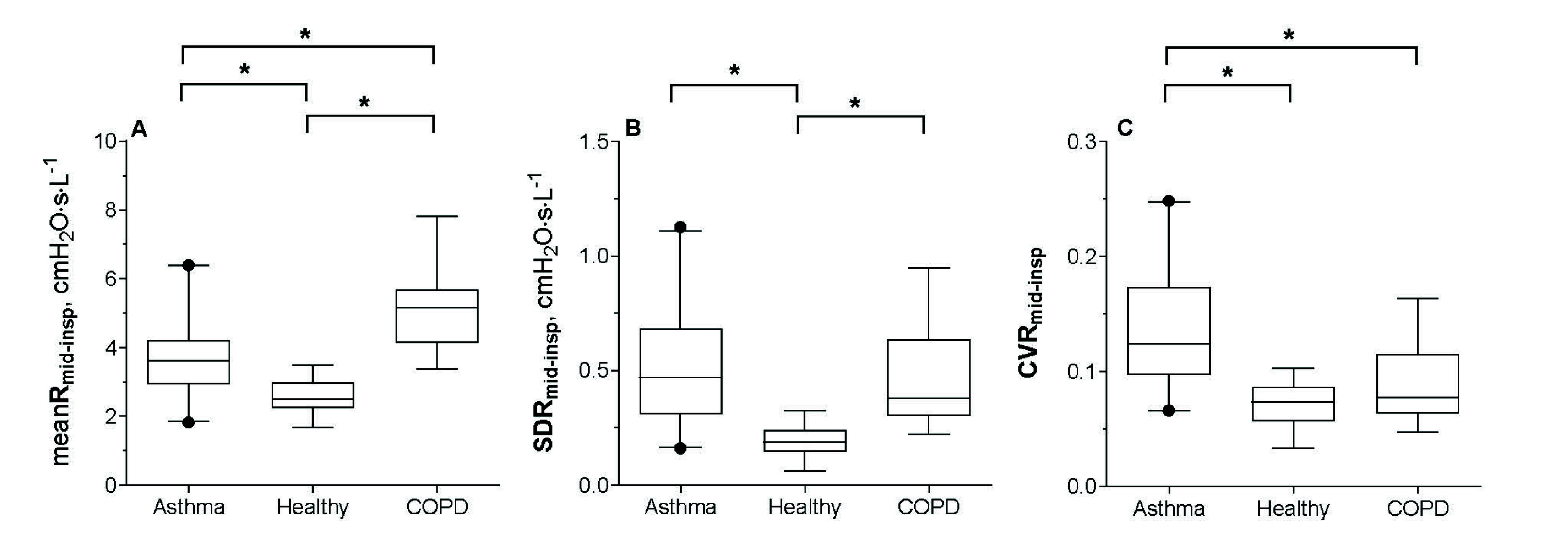
Distribution of meanR ${}_{\mathrm {mid-insp}}$ (panel A), SDR ${}_{\mathrm {mid-insp}}$ (panel B), and CVR ${}_{\mathrm {mid-insp}}$ (panel C) in Asthma, Healthy, and COPD subjects. Boxplots show the median, interquartile range, and 2.5th–97.5th percentile whiskers, with outliers displayed individually. Pooled data from development and validation phases. Symbols indicate statistically significant differences between groups (p<0.05), p-values vs Asthma are reported in the text. p-values for differences between Healthy and COPD were <0.0001 for meanR ${}_{\mathrm {mid-insp}}$ (panel A) and SDR ${}_{\mathrm {mid-insp}}$ (panel B), and 0.36 for CVR ${}_{\mathrm {mid-insp}}$ (panel C).

The meanR ${}_{\mathrm {mid-insp}}$ of asthmatic subjects was $3.72\pm 1.03$ cmH_2_O $\cdot $s $\cdot $L^−1^, significantly higher than in healthy ( $2.59\pm 0.50$ cmH_2_O $\cdot $s $\cdot $L^−1^, p<0.0001) but lower than in COPD ( $5.08\pm 1.09$ cmH_2_O $\cdot $s $\cdot $L^−1^, p= 0.0024) subjects (Figure 4A). SDR ${}_{\mathrm {mid-insp}}$ of asthmatic ( $0.51\pm 0.25$ cmH_2_O $\cdot $s $\cdot $L^−1^) was higher than in healthy ( $0.19\pm 0.06$ cmH_2_O $\cdot $s $\cdot $L^−1^, p<0.0001) subjects but not significantly different from COPD subjects ( $0.47\pm 0.23$ cmH_2_O $\cdot $s $\cdot $L^−1^, p>0.99) (Figure 4B). CVR ${}_{\mathrm {mid-insp}}$ of asthmatic ( $0.14\pm 0.05$) was significantly higher than either healthy ( $0.07\pm 0.02$, p<0.0001) or COPD ( $0.09\pm 0.04$, p= 0.0012) subjects (Figure 4C).

CVR ${}_{\mathrm {mid-insp}}$ was not different between males and females ( $0.120\pm 0.094$ vs $0.131\pm 0.100$, p= 0.89 in asthma; $0.073\pm 0.064$ vs $0.081\pm 0.060$, p= 0.60 in COPD; and $0.074\pm 0.016$ vs $0.069\pm 0.020$, p= 0.42 in healthy) and not associated with age in all groups (p= 0.73 in asthma; p= 0.13 in COPD; and p= 0.44 in healthy).

**FIGURE 5.**
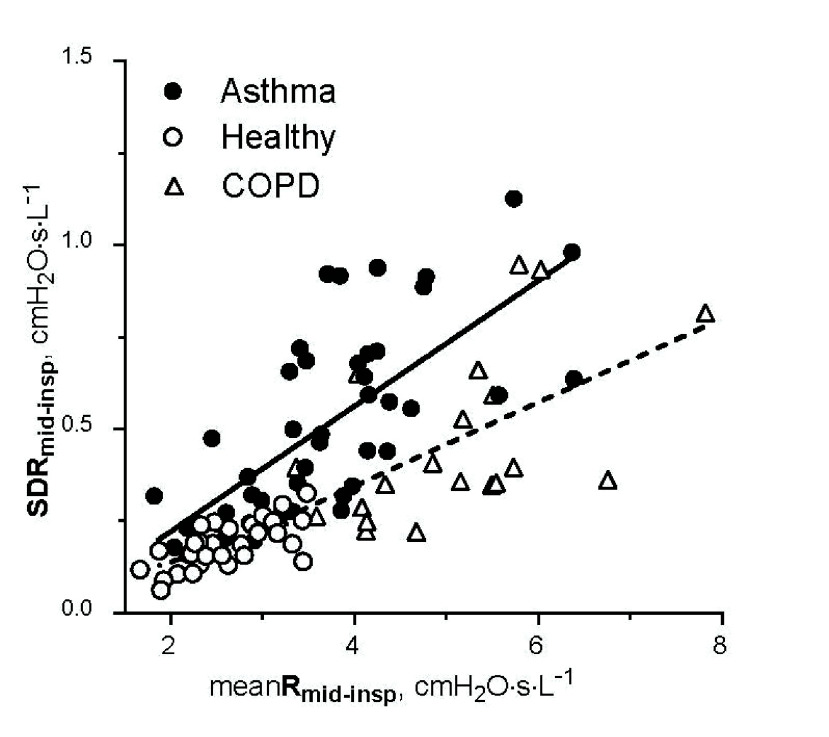
Relationship between SDR ${}_{\mathrm {mid-insp}}$ and meanR ${}_{\mathrm {mid-insp}}$ in asthmatic (full circles, solid line), healthy (open circles, dashed line), and COPD (triangles, interrupted line) groups. Pooled data from development and validation phases.

The SDR ${}_{\mathrm {mid-insp}}$ correlated linearly with meanR ${}_{\mathrm {mid-insp}}$ in asthmatic (r ${}^{2}=0.49$, p<0.001), healthy (r ${}^{2}=0.44$, p<0.001), and COPD (r ${}^{2}=0.30$, p<0.005) groups (Figure 5), while CVR ${}_{\mathrm {mid-insp}}$ was not correlated with meanR ${}_{\mathrm {mid-insp}}$ in any group.

The mean (95% confidence interval) accuracy (AUC) of CVR ${}_{\mathrm {mid-insp}}$ in separating asthmatics from subjects of the other groups was 0.86 (0.78–0.93). The optimal cutoff of 0.10 (0.09–0.10), resulted in 33 true positives, 9 false positives, 12 false negatives, and 44 true negatives. CVR ${}_{\mathrm {mid-insp}}$ provided a sensitivity of 0.73 (0.58–0.84), specificity of 0.83 (0.70–0.91), and diagnostic odds ratio of 12.74 (9.51–15.97). Excluding the subjects participating in the developmental phase of this study yielded similar results: AUC = 0.85 (0.75–0.93), sensitivity = 0.71 (0.55–0.84), and specificity = 0.88 (0.73–0.95).

## IV. Discussion

The main findings of the present study are that the day-to-day variability of respiratory resistance 1) was critically dependent on the portion of tidal breath that was sampled, 2) when calculated over mid-inspiration, provided an accurate separation of asthmatic from both healthy and COPD subjects, and 3) when expressed as coefficient of variation was not dependent on mean airway resistance.

Short-term variability of lung function has been long considered a characteristic feature of bronchial asthma [1], which is consistent with the variability of symptoms. For this reason, daily measurement of peak expiratory flow has been recommended to confirm diagnosis, as an alternative to bronchial challenges, and to monitor disease progression. In a pilot study [11], we found that the variability of respiratory resistance measured by oscillometry at home over a series of non-overlapping windows of four consecutive days each and averaged over the entire time-series separated stable asthmatic from healthy subjects and predicted the deterioration of lung function within the next 4-30 days. In a more recent study [6], we found that the accuracy in separating asthmatic from healthy subjects was higher with respiratory resistance than PEF. However, contrasting results have been reported by Timmins et al. [7], who did not find significant differences in the variability of supervised oscillometry measurements between stable asthmatic and healthy subjects over 7 non-consecutive days. Moreover, they found a significantly higher variability of inspiratory resistance in COPD than in healthy subjects, which is at odds with the stability of symptoms in COPD. Since mean resistance was higher in COPD than in asthmatic subjects, these findings were interpreted as suggesting that the variability of lung function represents a corollary to airway narrowing rather than a characteristic of asthma.

A major technical difference between Timmins et al. [7] and our studies [6], [11], was that they derived respiratory resistance using the whole inspiratory portion of each breath, whereas we used a mid-inspiratory portion only. In planning this study, we reasoned that the minimum observation time to differentiate groups might also depend on differences in intra-breath data sampling. Therefore, in the first phase of this study, we compared the time series of changes in respiratory resistance variability by sampling full breath, full inspiration, or mid-inspiration.

In the asthma group, we found CVR ${}_{\mathrm {mid-insp}}$ significantly higher than in both healthy and COPD groups at shorter time scales ( $\ge 7$ days) than either CVR ${}_{\mathrm {full-insp}}$ ( $\ge 9$ days) or CVR ${}_{\mathrm {full-breath}}$ (20 days). Moreover, CVR ${}_{\mathrm {full-insp}}$ was higher in asthmatic than healthy subjects at any time scale, which is at variance with the findings of Timmins et al. [7], who found no difference in either full breath or inspiratory resistance variability between asthmatic and healthy subjects at 7-day time scale. A possible explanation for this difference is that none of our asthmatic subjects were under regular treatment, whereas nine out of ten of their subjects were taking regular LABA or ICS or their combination.

In the COPD group, we found CVR ${}_{\mathrm {full-insp}}$ of COPD subjects significantly higher than in healthy subjects at time scales $\le 8$ days, which is consistent with Timmins et al.’s findings [7], but not at higher time scales. By contrast, CVR ${}_{\mathrm {mid-insp}}$ was not significantly different between COPD and healthy subjects at any time scale. All subjects in our study and nine out of ten in Timmins et. al. study [7] were on treatment with long-acting bronchodilators alone or in combination with inhaled corticosteroids.

Fluctuations in airway resistance in asthma have been attributed to fluctuations in smooth muscle contractile state that are faster and larger than in healthy subjects [8]. However, other factors may contribute to increasing the breath-to-breath variability of respiratory resistance. They include fluctuations in end-expiratory lung volume [22], differences between inspiratory and expiratory flow or tidal volume [23], upper airway changes in phase with the breathing cycle, dynamic airway compression, and expiratory flow limitation [24], [25], [26]. Altogether, these factors may represent a noise expected to decrease with increasing time scale, as happened in the present study. Sampling of mid-inspiratory phase excludes not only expiration but also the first portion of inspiration, where respiratory resistance may be affected by breath-to-breath changes in end-expiratory volume and airway closure or extreme airflow limitation occurring near the end of the prior expiratory phase. We interpret these findings as suggesting that the day-to-day variability of respiratory resistance reflects different mechanisms in asthma and COPD, depending on both data sampling and observation time.

Based on the results of the development phase, we validated the use of R ${}_{\mathrm {mid-insp}}$ for assessing day-to-day variability of lung function in the clinical diagnosis of asthma. CVR ${}_{\mathrm {mid-insp}}$ was significantly higher in the asthmatic than either healthy or COPD subjects, despite a significantly higher meanR_insp_ in the latter. This finding suggests that increased day-to-day variability of respiratory resistance expressed as CVR ${}_{\mathrm {mid-insp}}$ may be a characteristic feature of asthma independent of the presence or severity of airflow obstruction. This is confirmed by a stable, i.e., low-variance, optimal cutoff providing good-to-excellent accuracy, odds ratio, sensitivity, and specificity in distinguishing asthmatic from COPD and healthy subjects.

This study has some limitations. First, results are relative to the comparison of asymptomatic asthmatic subjects not receiving regular treatment with inhaled corticosteroids (ICS), or long-acting beta-agonists (LABA) during the monitoring phases, and COPD subjects on treatment. Thus, we cannot exclude that day-to-day variability of resistance might be different in untreated COPD or in asthmatics under regular treatment. Furthermore, the lack of data on pharmacological intake prevents evaluation of short-acting medication use among individuals with asthma and its potential confounding effect on the study outcomes. Further studies with larger sample sizes must evaluate the role of pharmacological treatments in the observed variability. This, however, cannot explain the differences in time series between CVR ${}_{\mathrm {full-breath}}$, CVR ${}_{\mathrm {full-insp}}$, and CVR ${}_{\mathrm {mid-insp}}$ nor invalidate the results on the relationship between CVR ${}_{\mathrm {mid-insp}}$ and airway narrowing. Second, the validation was not done in a separate sample but included the subjects of the development phase. The similarity in AUC between the development and validation phases makes us confident that this does not invalidate our conclusions. Third, the COPD group was not age-matched with other groups. However, age was not correlated with CVR ${}_{\mathrm {mid-insp}}$ and age differences cannot explain the differences between our study and the previous one in subjects with similar age differences [7]. Finally, future studies should address the impact of using higher stimulation frequencies combined with different data processing methods.

## V. Conclusion

In conclusion, the temporal variability of mid-inspiratory respiratory resistance assessed by daily self-administered oscillometry is a parameter specifically elevated in asthmatics compared to both healthy and COPD subjects. This data suggests that longitudinal oscillometry can provide clinically useful indexes of respiratory function for patient management, without requiring respiratory manoeuvres other than normal tidal breathing, thus resulting in good adherence, as confirmed by the present and previous longitudinal studies [6], [27].
